# Acceptance of Annual Booster Doses of COVID-19 Vaccines Among Indian Healthcare Professionals: A Pan-India Cross-Sectional Survey

**DOI:** 10.7759/cureus.49363

**Published:** 2023-11-24

**Authors:** Ekta Krishna, Venkatesh Karthikeyan, Shamshad Ahmad, Alok Ranjan, Abul Hasan KM, Sanjay Pandey, Pragya Kumar, CM Singh

**Affiliations:** 1 Community and Family Medicine, All India Institute of Medical Sciences, Patna, Patna, IND; 2 Pediatric Surgery, City Hospital Erode, Erode, IND; 3 Department of Community and Family Medicine, All India Institute of Medical Sciences, Patna, Patna, IND

**Keywords:** vaccine hesitancy, third booster dose, periodic dose, attitude, willingness, covid-19 vaccine, booster doses

## Abstract

Introduction

The emergence of coronavirus disease 2019 (COVID-19) posed significant challenges to global health, leading to the declaration of a pandemic by the World Health Organization. Vaccination efforts have effectively reduced severe outcomes and mortality, but breakthrough infections and new variants are of concern. In response, annual booster doses of COVID-19 vaccines are being considered to maintain immunity. Healthcare professionals, as frontline workers, play a pivotal role in vaccination campaigns. This study explores their attitudes toward and willingness to accept annual COVID-19 booster doses in India.

Methods

A pan-India cross-sectional survey was conducted among healthcare professionals, including faculty, resident doctors, interns, and nursing staff, across Indian medical and nursing colleges. Convenience sampling was used to collect responses via an online questionnaire. The questionnaire assessed demographics, vaccine status, attitudes toward COVID-19 vaccination, and willingness to accept annual booster doses. Multivariate analysis was performed to identify predictors of booster dose acceptance.

Results

A total of 535 participants responded from 28 states and 8 union territories of India. Most were 34.2 years (± 11.1 SD), and 372 (69.5%) had taken Covishield (Serum Institute of India, Pune, India) as their primary vaccine. While 525 (98.1%) had taken the first dose and 518 (96.8%) of them had taken the second dose, only 333 (62.2%) had received a booster. Around 318 (60%) of healthcare professionals were willing to accept an annual booster dose. The mean attitude score toward annual booster doses was 75.4 (range: 28-111). Healthcare professionals' trust in government recommendations and medical experts significantly influenced their willingness to accept annual booster doses.

Conclusion

This study provides insights into the attitudes of healthcare professionals in India toward annual COVID-19 booster doses. At the same time, a significant proportion showed a willingness to accept boosters.

## Introduction

The coronavirus disease 2019 (COVID-19) pandemic began in Wuhan, China, and rapidly spread worldwide, leading to countless deaths and significant global health challenges [1]. On January 30, 2020, the World Health Organization (WHO) declared it an international health emergency, and by March 11, 2020, it had escalated to a pandemic [2]. This pandemic has resulted in immense loss of life and economic hardship on a global scale. The introduction of COVID-19 vaccines globally has played a vital role in reducing the severity of illnesses, mortality rates, and hospitalizations caused by the severe acute respiratory syndrome coronavirus 2 (SARS‑CoV‑2) virus. However, SARS-CoV-2 continues to circulate and remains a leading cause of breakthrough infections. These breakthrough infections are largely attributed to a weakening immune response following natural infection or vaccination, as well as the evasion of immunity by emerging variants of concern [3,4]. In December 2021, India witnessed a rapid surge in Omicron cases in major urban areas. Subsequently, in 2022, India faced another severe wave of the pandemic driven by the Delta variant [5]. Despite robust vaccination efforts using Covaxin (Bharat Biotech, Hyderabad, India) and Covishield (Serum Institute of India, Pune, India), the country reported cases of breakthrough infections and re-infections [6]. Observations from other nations highlighting the benefits of additional vaccine doses to enhance immunity prompted India to initiate booster dose vaccinations in January 2022. Although widespread COVID-19 vaccination has contributed to achieving substantial herd immunity, multiple studies indicate the potential for declining immunity and the emergence of new SARS-CoV-2 variants [7]. Transmission risks are particularly pronounced among individuals with weakened immune systems, such as the elderly and those with chronic illnesses [8]. Given the persisting occurrence of COVID-19 cases in various regions, an effective strategy to break the transmission chain is the introduction of annual booster doses targeting different vulnerable age groups. Such booster doses hold the potential not only to reinforce existing herd immunity but also to mitigate the mortality and morbidity associated with potential future outbreaks. Healthcare professionals, including doctors and nursing staff working in hospitals, face elevated risks of infection during COVID-19 waves. Hence, this quantitative study seeks to know the perspectives of healthcare professionals regarding the incorporation of annual booster doses for COVID-19 vaccines and their willingness to embrace such booster doses.

## Materials and methods

Study design, setting, and study population

A descriptive cross-sectional study was undertaken to investigate the acceptance and attitudes of healthcare professionals, including faculty and resident doctors, as well as interns and Nursing staff, across various Medical and Nursing colleges within India. The study was conducted over a span of two months, from May 1, 2023, to June 30, 2023.

Sample size and sampling procedure

To determine the required sample size, the prevalence of individuals in the general population willing to receive periodic (annual) doses of the COVID-19 vaccine was derived from a previous study by Abuhammad et al. [7], wherein the prevalence was found to be 19.3% with a 95% confidence interval and an absolute precision of 5%. Sample size calculations employed a formula for single proportion estimation using the Statulator web-based software (https://statulator.com/). The calculated minimal sample size was 237. However, a total of 535 responses were collected over the two-month period. Convenience sampling, a non-probability sampling technique, was utilized to gather the study participants. Convenience sampling allowed us to efficiently collect responses within a limited timeframe and resource availability. Additionally, we encountered challenges in accessing comprehensive and up-to-date databases that encompassed a diverse range of healthcare professionals across various states. Given these constraints, convenience sampling was deemed a reasonable approach to gather a representative sample of healthcare professionals willing to participate in our survey.

Data collection procedures

Data were collected through an online survey using a pre-designed, pre-validated questionnaire hosted on Google Forms. The survey link was widely disseminated via various social media platforms, including Facebook, WhatsApp, and email. The investigators shared the survey link through individual messages, WhatsApp groups, and email IDs. Participant confidentiality was rigorously maintained throughout the study. A consent form was provided at the outset of the survey, clearly outlining the study's purpose and nature. The questionnaire encompassed three main sections: 1) Socio-demographic characteristics, 2) Acceptance of healthcare professionals for annual booster doses, and 3) Attitudes of healthcare professionals toward COVID-19 vaccination. The third section, containing 24 items, gauged participant attitudes using a scoring scale ranging from strongly disagree (1) to strongly agree (5) for positive attitude items and vice versa for negative attitude items. This attitude questionnaire was adapted from the work of Abuhammad et al., which assessed public attitudes toward periodic COVID-19 vaccination [7]. Total scores ranged from 24 to 120, with higher scores indicating a more favorable attitude toward receiving periodic vaccine doses.

Statistical analysis

Data were initially exported from Google Sheets to an Excel spreadsheet for preliminary cleaning and coding. The analysis was then conducted using Jamovi version 2.3.28 (https://www.jamovi.org/download.html). Descriptive statistics, including mean and standard deviation for quantitative variables like age and attitude score, and percentages for categorical variables such as gender, religion, occupation, willingness to receive annual booster COVID-19 vaccines, etc., were reported. The relationship between introducing and accepting annual booster doses of COVID-19 vaccines and independent variables was explored using multiple linear regression. Univariate analysis was initially performed, and variables with a p-value ≤ 0.2 were considered for multivariate analysis. The normality of residuals was checked by an inspection of the histogram and P-P plot. Linearity was assessed by partial regression plots and a plot of studentized residuals against the predicted values. Homoscedasticity is assessed by visual inspection. The significance tests for all variables were conducted at a p-value threshold of < 0.05.

Ethical considerations

The study protocol received ethical approval from the Ethical Committee of AIIMS, Patna (Ref. No. AIIMS/Pat/lEC/2022/1052). Informed consent was secured verbally after providing participants with a comprehensive understanding of the study's objectives. Voluntary participation was emphasized, and written informed consent was obtained from participants before proceeding with the main section of the questionnaire.

## Results

Demographic characteristics of study participants

**Figure 1:**
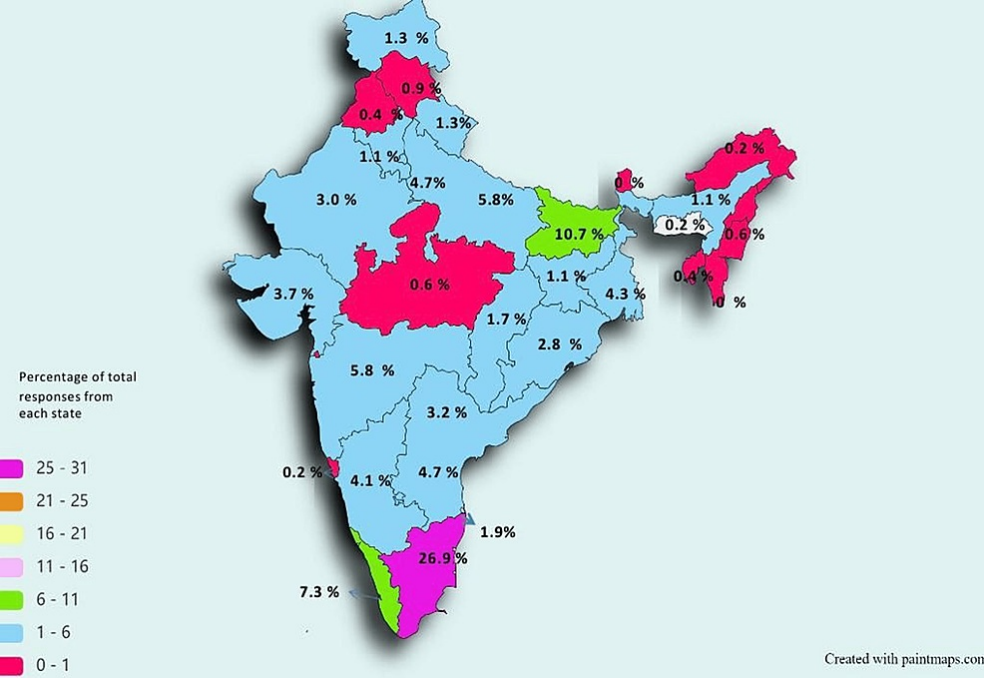
State/Union territory-wise participation of healthcare professionals of India in the survey (N = 535)

A total of 20, 000 healthcare professionals were approached originally, where 535 healthcare professionals gave consent to take part in this study survey. So, the response rate obtained for this study survey is around 3 %. The highest response rates were observed in Tamil Nadu 144 (26.9%), Bihar 57 (10.7%), Uttar Pradesh 31 (5.8%), and Maharashtra 31 (5.8%). Figure 1 illustrates the distribution of responses from other states.

The mean age of study participants was 34.2 years ± 11.1 (SD) years. Gender distribution was nearly equal, with almost an equal number of male and female participants. Most participants (408, 76.3%) hailed from nuclear families. In terms of professional roles, 189 (35.3%) were faculty members, followed by junior residents 168 (31.4%), senior residents 73(13.6%), MBBS students 41 (7.7%), and interns 39 (7.3%). Nursing staff and students constituted 25 (4.6%) out of the total participants. A significant portion of 272 (50.8%) reported an income exceeding 1 lakh. Furthermore, 294 (55%) of respondents reported having a family member aged 65 years or older, and 80 (15%) disclosed having non-communicable diseases (NCDs) such as diabetes, hypertension, and chronic obstructive pulmonary disease (COPD). A majority of participants 287 (53.6%) actively used two or more social media platforms (Table 1).

**Table 1:** Demographical characteristics of the participants (N = 535)

Variables	Categories	Frequency (n)	Percentage (%)
The mean age of study participants (34.2 ± 11.1) (SD)			
Age (in years)	<20	6	1.10%
	20-30	229	42.80%
	>30	300	56.10%
Gender	Female	271	50.7 %
	Male	264	49.3%
Religion	Hindu	440	82.2 %
	Christian	49	9.2%
	Muslim	24	4.5 %
	Others	22	4.1%
Type of Family	Nuclear	408	76.3 %
	Joint	127	23.7%
Occupation	Faculty	189	35.3 %
	Junior Residents	168	31.4%
	Senior Residents	73	13.6%
	MBBS Students	41	7.7 %
	Intern	39	7.3%
	Nursing staff & students	25	4.6%
Monthly income of the family (in rupees)	Less than 50,000	109	20.4 %
	50, 000-1,00,000	154	28.8%
	>1,00, 000	272	50.8%
Marital status	Married	274	51.2 %
	Unmarried	244	45.6 %
	Others (Widow/separated/divorced)	17	3.10%
Do you have a family member aged 65 years or older	Yes	294	55.0 %
	No	241	45.0%
Are you suffering from Non-communicable diseases (NCDs) like cardiovascular diseases (CVDs), diabetes, hypertension, chronic obstructive pulmonary disease (COPD), and cancer?	Yes	80	15.0 %
	No	455	85.1%
Use of social media	0-1	143	26.7%
	2 to 3	287	53.6%
	>3	105	19.6%

COVID-19 vaccine status of participants

**Figure 2:**
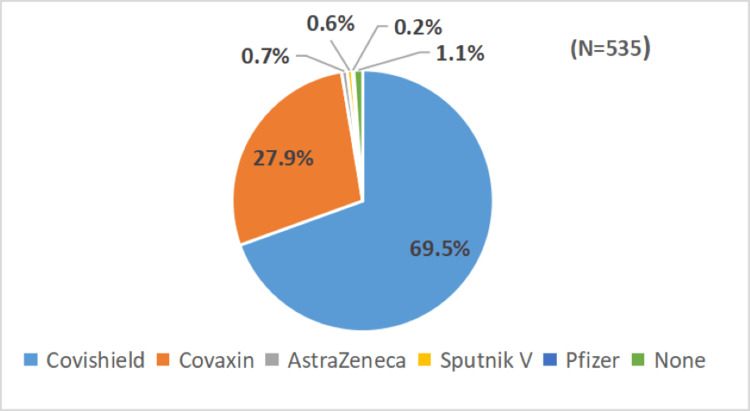
Various COVID-19 vaccines taken by study participants

Of the healthcare professionals, 525 (98.1%) had received the first dose of the COVID-19 vaccine, and 518 (96.8%) had received the second dose. In contrast, a comparatively lower proportion (333, 62.2%) had taken a booster dose of the COVID-19 vaccine (Table 2). Among the vaccine options, 372 (69.5%) of respondents had received the Covishield vaccine while 149 (27.9%) had received the Covaxin vaccine. A smaller proportion had received AstraZeneca - 4 (0.7%), Sputnik V -3 (0.6%), and Pfizer 1 - (0.2%) (Figure 2).

**Table 2:** COVID-19 vaccine status of the participants (N=535)

	Categories	Frequency (n)	Percentage (%)
)Have you taken the first dose of COVID-19 vaccine?	Yes	525	98.1 %
	No	10	1.9 %
Have you taken a second dose of COVID-19 vaccine?	Yes	518	96.8 %
	No	17	3.2 %
Have you taken a booster dose of the COVID-19 vaccine?	Yes	333	62.2%
	No	202	37.8%

Willingness for annual booster doses

**Figure 3:**
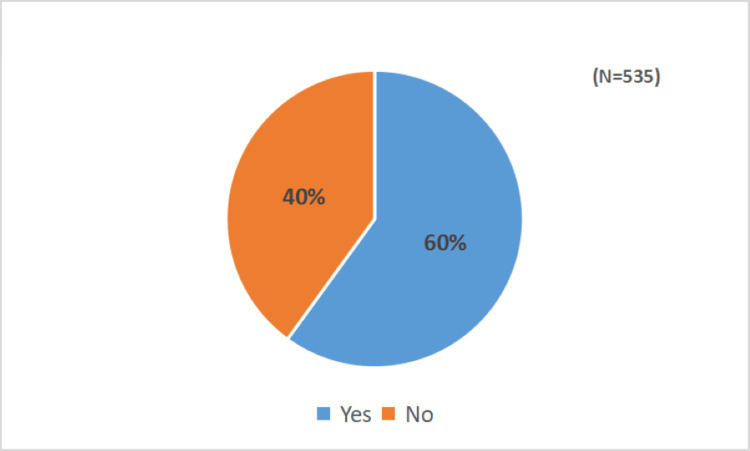
Willingness to take an annual booster dose of the COVID-19 vaccine among study participants

Out of 535 respondents, 333 (62.2%) of participants reported receiving the COVID-19 vaccine booster dose. Interestingly, most of the respondents (318, 60%) who had received a booster dose of the COVID-19 vaccine showed willingness to take an annual booster dose of the COVID-19 vaccine (Figure 3).

Healthcare professionals’ attitudes toward periodic COVID-19 vaccination

**Figure 4:**
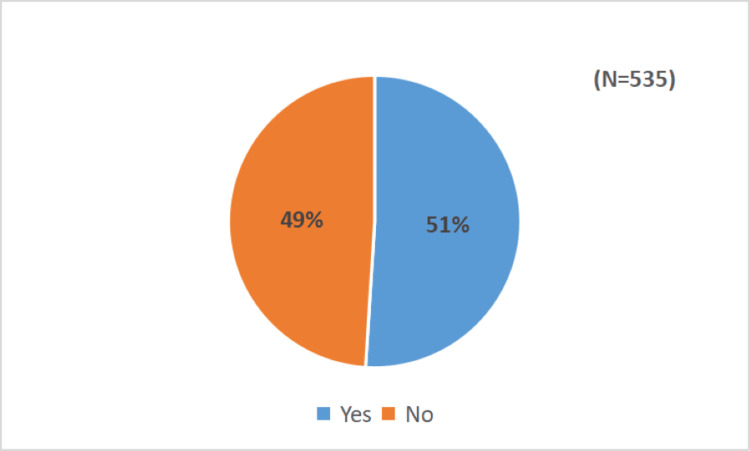
Proportion of participants with a positive attitude toward the introduction of an annual booster dose of the COVID-19 vaccine

The mean attitude score of study participants toward the introduction of annual booster doses of the COVID-19 vaccine was notably high at 75.4 (range: 28-111). Around 273 (51%) healthcare professionals scored higher than the mean attitude score, reflecting a favorable outlook on the annual booster doses. Figure 4 illustrates the distribution of scores. Noteworthy "agree" responses were observed for the following attitude components: the protective efficacy of the annual booster dose (228, 42.6%), potential regret for not receiving the booster (239, 44.7%), ease of obtaining the annual booster dose (315, 58.9%), concerns about side effects (222, 41.5%), potential regret after experiencing side effects (248, 46.4%), confidence in the newness of the annual booster (228, 42.6%), the anticipation of others receiving the booster (242, 45.2%), overall positivity toward vaccination (437, 88.4%), informed decision-making (373, 69.7%), informed decision about the COVID-19 vaccines (363, 67.9%), approval of the booster by family (268, 50.1%), and endorsement by friends (228, 42.6%). Notably, 358 (66.9%) of participants indicated trust in government recommendations by agreeing to receive the booster, and a similar proportion would recommend the booster to others if endorsed by experts. For additional variables concerning healthcare professionals' attitudes toward periodic COVID-19 vaccination (Table 3).

**Table 3:** Description of Indian healthcare professional's attitudes toward receiving periodic doses of COVID-19 vaccine (N=535)

Questions	Categories	Frequency (n)	Percentage (%)
Annual booster doses of COVID-19 must be made mandatory for every person who can receive them	AGREE	227	42.4 %
	DISAGREE	163	30.5 %
	NEUTRAL	145	27.1 %
Without a COVID-19 periodic vaccination, I would probably have contracted COVID-19	AGREE	211	39.4 %
	DISAGREE	178	33.3 %
	NEUTRAL	146	27.3 %
The annual booster dose of COVID-19 will protect me from COVID-19	AGREE	228	42.6 %
	DISAGREE	141	26.4 %
	NEUTRAL	166	31.0 %
If I get infected by COVID-19 without taking the annual booster dose of the COVID-19 vaccine, I might regret not getting the booster vaccination	AGREE	239	44.7 %
	DISAGREE	181	33.8 %
	NEUTRAL	115	21.5 %
It would be very easy for me to get the annual booster dose of the COVID-19 vaccine	AGREE	315	58.9 %
	DISAGREE	79	14.8 %
	NEUTRAL	141	26.4 %
The annual booster dose of COVID-19 may infect me with the virus	AGREE	70	13.1 %
	DISAGREE	347	64.9 %
	NEUTRAL	118	22.1 %
I would be worried about suffering from the side effects of an annual booster dose of COVID-19 vaccination	AGREE	222	41.5 %
	DISAGREE	195	36.4 %
	NEUTRAL	118	22.1 %
I may regret receiving ng annual booster dose of the COVID-19 vaccine if I later experience side effects from the vaccination	AGREE	248	46.4 %
	DISAGREE	161	30.1 %
	NEUTRAL	126	23.6 %
The annual booster dose of COVID-19 would be too new for me to be confident about getting vaccinated	AGREE	228	42.6 %
	DISAGREE	172	32.1 %
	NEUTRAL	135	25.2 %
Most people will receive an annual booster dose of COVID-19 vaccination	AGREE	179	33.5 %
	DISAGREE	206	38.5 %
	NEUTRAL	150	28.0 %
Other people like me will receive an annual booster dose	AGREE	242	45.2 %
	DISAGREE	135	25.2 %
	NEUTRAL	158	29.5 %
In general, vaccination is a good thing	AGREE	473	88.4 %
	DISAGREE	24	4.5 %
	NEUTRAL	38	7.1 %
I am afraid of needles	AGREE	73	13.6 %
	DISAGREE	396	74.0 %
	NEUTRAL	66	12.3 %
If I get an annual booster dose of COVID-19, I think I will not need to follow the social distancing and other restrictions imposed due to COVID-19	AGREE	94	17.6 %
	DISAGREE	362	67.7 %
	NEUTRAL	79	14.8 %
I know enough about COVID-19 to make an informed decision	AGREE	373	69.7 %
	DISAGREE	47	8.8 %
	NEUTRAL	115	21.5 %
I know enough about the COVID-19 vaccines to make an informed decision about whether to get an annual booster dose of COVID-19 vaccine	AGREE	363	67.9 %
	DISAGREE	48	9.0 %
	NEUTRAL	124	23.2 %
Only people at risk of COVID-19 need a periodic dose of vaccination	AGREE	199	37.2 %
	DISAGREE	224	41.9 %
	NEUTRAL	112	20.9 %
My family will approve the annual booster dose of COVID-19 vaccination	AGREE	268	50.1 %
	DISAGREE	119	22.2 %
	NEUTRAL	148	27.7 %
My friends will approve annual booster dose of COVID-19 vaccination	AGREE	228	42.6 %
	DISAGREE	116	21.7 %
	NEUTRAL	191	35.7 %
If the government recommends the annual booster dose of COVID-19 vaccination, I will get vaccinated	AGREE	358	66.9 %
	DISAGREE	80	15.0 %
	NEUTRAL	97	18.1 %
If a healthcare professional recommends annual booster periodic vaccination for COVID-19, I will get vaccinated	AGREE	358	66.9 %
	DISAGREE	78	14.6 %
	NEUTRAL	99	18.5 %
The annual booster dose of COVID-19 vaccination is just a way for vaccine manufacturers to make money	AGREE	148	27.7 %
	DISAGREE	199	37.2 %
	NEUTRAL	188	35.1 %
The annual booster dose of COVID-19 vaccination will allow us to return to normal life	AGREE	174	32.5 %
	DISAGREE	199	37.2%
	NEUTRAL	162	30.3%
There will be no point in getting the annual booster dose of the COVID-19 vaccine unless I can return to my normal life	AGREE	194	36.3%
	DISAGREE	166	31.0%
	NEUTRAL	175	32.7%

Predictors of attitudes toward annual vaccine doses

First, univariate analysis was done where linear regressions were run for each independent variable to find out its association with the Attitude score without doing any adjustments in other variables. Then, multiple linear regression (MLR) was run to predict attitude scores from age, marital status, use of multiple social media, uptake of the first COVID-19 vaccine dose, and booster dose uptake (Table 4). Only uptake of a booster dose of COVID-19 added significantly to the prediction (p<0.05).

**Table 4:** Predictors of attitudes toward receiving periodic doses of the COVID-19 vaccine (N=535) Ref-Reference category, * p<0.05

Predictors	Univariate Analysis		Multivariate Analysis			
	B-coefficient	p	B-coefficient	Lower	Upper	p
Age (in years)	-0.094	0.025	-0.0668	-0.172	0.0384	0.213
Gender: Male-Female (Ref)	1.08	0.247				
Religion: Hindu-Other (Ref)	1.52	0.211				
Type of Family: Nuclear-Joint (Ref)	0.5	0.647				
Marital status: Unmarried-Married (Ref)	1.95	0.038	1.1935	-1.048	3.434	0.296
Others-Married (Ref)	-4.68	0.08	-3.1176	-8.444	2.208	0.251
Do you have a family member of age 65 years or older? Yes-No (Ref)	-1.05	0.259				
Living Area: Urban-Rural (Ref)	-0.0854	0.943				
Did you suffer COVID-19 in the past? Yes-No (Ref)	-1.08	0.248				
Are you suffering from any non-communicable diseases (NCDs) like cardiovascular diseases (CVDs), diabetes, hypertension, chronic obstructive pulmonary disease (COPD), and cancer? Yes-No (Ref)	-1.18	0.365				
Number of social media used	0.612	0.14	0.3515	-0.473	1.176	0.403
Have you taken the first dose of the COVID-19 vaccine? Yes-No (Ref)	7.56	0.027	5.6582	-1.093	12.409	0.100
Have you taken the second dose of the COVID-19 vaccine? Yes-No (Ref)	2.56	0.334				
Have you taken a Booster dose of the COVID-19 Vaccine? Yes-No (Ref)	2.47	0.01	2.5129	0.587	4.438	0.011*

## Discussion

Overall, half of the healthcare professionals in India have a good attitude toward the introduction of the annual booster of the COVID-19 vaccine. This study found that three-fifths of Indian healthcare professionals have shown a willingness to take annual booster doses of the COVID-19 vaccine. In neighboring countries like Bangladesh and China, the acceptance rate for booster doses among healthcare professionals is seen to be 84.6% and 76.8%, respectively [9,10]. In other nations like the USA, Czechia, Poland, and UAE, the intention to accept the booster dose of the COVID-19 vaccine among healthcare professionals was found to be 83.6 %, 71.3%, 74.5, and 70.2%, respectively [11-14]. According to the Health Believe model, self-perceived risk of infection is related to one's vaccination intention [15,16]. Similar results were found in our study where vaccination intention to get a booster dose is higher among healthcare professionals when compared with the general public of India [17].

In our study, coverage of primary, secondary, and booster doses of the COVID-19 vaccine among healthcare professionals in India was found to be 98.1%, 96.8%, and 62.2%, respectively. Similarly, in another study conducted among a large adult population of Delhi, it was found that 95.1% had received two primary doses of the COVID-19 vaccine while 58.28% had received a COVID-19 booster dose vaccine along with two primary doses of the COVID-19 vaccine [18]. Likewise, as of August 4, 2023, the Co-WIN dashboard showed out of the total who had received the first doses of the COVID-19 vaccine, 92.6% of them had received the second dose of COVID-19 vaccine while a very low percentage (22.1%) of people had received a booster dose of the COVID-19 vaccine [19]. So, these findings further suggest uptake of booster doses is poor among the general population compared to healthcare professionals.

The majority of the healthcare professionals (69.5%) in our study had taken Covishield while 27.5% of them had taken Covaxin. Similar findings were observed in studies conducted by Rahi M et al. [20] and Masthi NR et al. [13], where more than four-fifths of participants had taken Covishield while a few of them had taken Covaxin. The reason behind this could be that Covishield was the first vaccine that was launched in India among front-line workers, including doctors, and another reason includes its effectiveness being seen to be more than Covaxin as seen in many studies [21-23].

Generally, it has been observed in previous studies that the common driving force for COVID-19 vaccine uptake is health literacy regarding the vaccine and its effectiveness, accessibility to the vaccine, trust in the government, fear of suffering from adverse effects after taking the vaccine, economic vulnerability, misinformation/ infodemic, etc. [9,24,25].

The major portion of the healthcare professional in our study is confident regarding their knowledge of COVID-19 and the COVID-19 vaccine to make an informed decision on it as depicted in Table 4. Around half of them also agreed that even their family members and friends would also take the annual booster if given a choice. Despite the high confidence in the COVID-19 vaccine, our studies showed only 62.2% of healthcare professionals have taken a booster dose of the COVID-19 vaccine [19].

Two-fifths of healthcare professionals recommend an annual booster dose of the COVID-19 vaccine for the most vulnerable groups like the elderly and immune-compromised. The reason behind this recommendation could be the waning immunity after the third dose of COVID-19 [26,27]. A study by Gilboa et al. also showed waning immunity is much more among the elderly aged >65 years, which further supports our study findings and highlights a further need for the introduction of an annual booster COVID-19 vaccine for special groups [27].

Our survey further highlighted the role of medical experts’ opinions and government recommendations of a vaccine are among the major key enablers in building positive attitudes towards beneficiaries [28,29]. Being a prioritized group, three-fifths of healthcare professionals in India agreed that they won’t face problems in vaccine accessibility.

Our study provided evidence that healthcare professionals are fully aware of the fact that the COVID-19 vaccination can reduce serious mortality and morbidity but not give complete protection against new infections [30]. Around 7 out of 10 healthcare professionals in India who participated in this survey didn’t agree that after taking the annual booster of the COVID-19 vaccine, they won’t be required to follow prevention measures like social distancing and two-fifths of them disagreed that getting an annual booster dose will help us in returning to normal life.

This study revealed on MLR that the uptake of the first booster of the COVID-19 vaccine is significantly associated with attitude score for receiving an annual booster dose of the COVID-19 vaccine. In another study conducted in India, the willingness to take a booster dose of the COVID-19 vaccine is found to be significantly associated with gender, lower income, lower educational attainment, rural-urban residence, completion of primary shots of the COVID-19 vaccine, and family and friends never infected by SARS-CoV-2 virus. In a study conducted in Ethiopia with a similar objective, using a similar instrument among the general public, factors that are seen to be significantly associated with attitude scores for receiving periodic doses of the COVID-19 vaccine are income, educational attainment, and news related to COVID-19 vaccine [8]. In an online survey conducted in the US, major determinants affecting COVID-19 vaccine acceptance among the general public are marital status, income status, and employment [31].

Strengths

This is one of the first studies conducted in India that has tried to know the intention of healthcare professionals toward the introduction and acceptance of the annual booster dose of the COVID-19 vaccine. Healthcare professionals who participated in this study were from all over the country. 

Limitations

This survey was conducted over a short period of two months (May-June). Our survey represents a snapshot of attitudes among a subset of healthcare professionals rather than a comprehensive study of the entire population. The large population of healthcare professionals in India makes it challenging to achieve a fully representative sample. Most notably, data collection was done during a period when no outbreak happened. So if the study was conducted over a large interval of time, a time trend analysis could have been done. The sampling method used in this study was convenience sampling, so the responses obtained from different states were not the same. Due to the limited sample size and the observed low response rates in some states (less than 1%), it was not feasible to conduct meaningful state-wise comparisons with the available data. The data was collected using a self-administered questionnaire, which might have resulted in some bias in the study due to self-reporting.

## Conclusions

This study provides insights into the attitudes of healthcare professionals in India toward annual COVID-19 booster doses. While a significant proportion showed a willingness to accept boosters, further research and awareness campaigns are needed to increase acceptance rates, especially among this essential group. The findings emphasize the role of healthcare professionals in shaping public vaccination behaviour and recommend targeted strategies for promoting booster doses.
